# The opsin repertoire of *Jenynsia onca*: a new perspective on gene duplication and divergence in livebearers

**DOI:** 10.1186/1756-0500-2-159

**Published:** 2009-08-05

**Authors:** Diana J Windsor, Gregory L Owens

**Affiliations:** 1 Department of Biology, University of Victoria, 3800 Finnerty Rd, Victoria, British Columbia, Canada

## Abstract

**Background:**

*Jenynsia onca*, commonly known as the one sided livebearer, is a member of the family Anablepidae. The opsin gene repertoires of *J. onca*'s close relatives, the four-eyed fish (*Anableps anableps*) and the guppy (*Poecilia reticulata*), have been characterized and each found to include one unique LWS opsin. Currently, the relationship among LWS paralogs and orthologs in these species are unclear, making it difficult to test the hypotheses that link vision to morphology or life history traits. The phylogenetic signal appears to have been disrupted by gene conversion. Here we have sequenced the opsin genes of *J. onca *in order to resolve these relationships.

**Findings:**

We identified nine visual opsins; LWS S180r, LWS S180, LWS P180, SWS1, SWS2A, SWS2B, RH1, RH2-1, and RH2-2. Key site analysis revealed only one unique haplotype, RH2-2, although this is unlikely to shift λ_max _significantly. LWS P180 was found to be a product of a gene conversion event with LWS S180, followed by convergence to a proline residue at the 180 site.

**Conclusion:**

*Jenynsia onca *has at least 9 visual opsins: three LWS, one RH1, two RH2, one SWS1 and two SWS2. The presence of LWS P180 moves the location of the LWS P180-S180 tandem duplication event back to the base of the Poeciliidae-Anablepidae clade, expanding the number of species possessing this unusual blue shifted LWS opsin. The presence of the LWS P180 gene also confirms that gene conversion events have homogenized opsin paralogs in fish, just as they have in humans.

## Background

*Jenynsia *is the sister group to the genus *Anableps*, together they form the subfamily Anablepinae; the genus *Oxyzygonectes *is the sister group of the subfamily Anablepinae and jointly they form the family Anablepidae. The genus *Jenynsia *is comprised of thirteen species [1]. *Jenynsia onca *is distributed in freshwater lakes and rivers throughout southern Brazil [1]. *J. onca *is a pelagic freshwater fish, whose eyes are morphologically normal in appearance, unlike *A. anableps *a member of it's sister genus [2]. Distinguishing features of a male *J. onca *include a tubular unscaled gonopodium, which is either dextral or sinistral. Females of this species correspondingly have either a dextral or sinistral genital opening and mate only with complementary sided males. Additionally, *J. onca *has dark circular spots on the ventral portion of the flank [1,2]. Here we have used PCR to characterize the opsin genes of this species.

Vision is an interesting and dynamic sensory modality, particularly in teleost fish, a group which possess some of the greatest morphological and habitat diversity of any animal group. The first step of vision is light absorption, which occurs via opsins expressed in the rods and cones. There are five sub families of vertebrate opsins each with their maximal absorption (λ_max_) focused on a different area of the visual spectrum [3]: RH1 a rod specific class (490-500 nm), LWS a long-wave sensitive class (490-570 nm), RH2 a middle-wave sensitive class (480-533 nm), SWS1 and SWS2 short-wave sensitive classes (355-440 nm and 410-490 nm respectively) [4]. Within these five classes of conserved opsin proteins, there are variable amino acid residues that give each opsin its unique spectral sensitivity [4-10]. These residues are termed key sites, they are often found at positions in which contact is made with the chromophore, and each has a disproportionate effect on the λ_max _[5,11].

Opsin repertoires in fish are particularly interesting due to the extensive pattern of opsin gene duplication and divergence found throughout teleostei. Furthermore, the expanded repertoire is often correlated to differential expression, both spatially across the retina and developmentally over time. For example, in zebrafish (*Danio rerio*) it has been demonstrated that LWS duplicates localize differentially across the topology of the retina, and that this pattern of expression also changes during development [12]. This has been hypothesized to be a response to the heterogeneous environment the fish live in, with spectral properties differing between the light that hits the dorsal region of the retina and that which hits the ventral region. Another example of differential expression is in cichlids, in this system a subset of the available opsin repertoire is used by each particular species to tune sensitivity in a habitat dependent way [13]. The first step to characterizing these fascinating expressional patterns is through the elucidation of the opsin repertoire itself.

Particularly interesting are the opsin repertoires of the livebearers; ongoing studies of opsin gene duplication and divergence in guppies (*Poecilia reticulata*) and the four-eyed fish (*Anableps anableps*) have shown that both species have an expanded LWS repertoire. Both species have recent species-specific duplicates and a repertoire of ten visual opsins [14-16]. Interestingly both also have remarkable morphology, in the case of the guppy this is a variable male pigmentation pattern, while *A. anableps *have unusual four-eyed morphology. Based on its phylogenetic position, *J. onca *functions as a useful out-group for comparison of opsin repertoire, particularly between *A. anableps *and the Poeciliids [17]. Out-groups are used to identify synapomorphies (shared derived characters) and can indicate whether one apomorphic trait evolved before another. Here our goal is to determine whether or not particular opsin genes and gene sequences are associated with coloration in guppies and the four-eye morphology in *A. anableps*.

## Results and Discussion

PCR screening using gene specific primers (Table 1) identified nine visual opsins: LWS S180r, LWS S180, LWS P180, SWS1, SWS2A, SWS2B, RH1, RH2-1, & RH2-2. All opsins except LWS S180r and S180 are expressed, having been amplified from eye cDNA derived from one adult male and one adult female *J. onca*. LWS S180r and S180 opsins were amplified only from genomic DNA. However the reason that LWS S180 and S180r could not be retrieved from cDNA could be attributed to life stage, as only adults were used in this study. A particularly interesting finding in this repertoire was the LWS P180 opsin, as it's presence moves the point of LWS duplication farther back within the Cyprinodontiformes order. We did not find any species-specific gene duplication events in *J. onca*.

**Table 1:** Primers used for *J. onca *cDNA and genomic PCR.

Opsin category	Primer Name	Sequence
SWS1	SWS1Fw1	5'-AACTACATCYTGGTMAACATCTCC-3'
	
	SWS1Rev2	5'-GAACTGTTTGTTCATGAAGGCG-3'

SWS2	SWS2Fw1	5'-GYACWATTCAATACAAGAARC-3'
	
	SWS2Rev2	5'-TCTCWGCCTTCTGGGTKGAGGC-3'
	
	SWS2AFw1	5'-GTCCACCCGAGTCATAGAGC-3'
	
	SWS2ARev2	5'-GCCCACGGTTGTTGACAAC-3'

RH2	RH2Fw1	5'-AACTTCTAYATCCCGWTGTCC-3'
	
	RH2Rev1	5'-AGCATGCAGTTACGGACTG-3'
	
	RH2-2Fw1	5'-CAACAGGACGGGCTGGTGAGG-3'
	
	RH2-2Rev3	5'-ACCCATTCCAATTGTTGCC-3'

RH1	RH1Fw2	5'-GGAGTCCTTATGAATATCCTCAG-3'
	
	RH1Rev2	5'-CCTGTTGCTCCATTTATGCAGG-3'

LWS	Fw100	5'-GATCCCTTTGAAGGACCAAACT-3'
	
	Fw1a	5'-TCTTATCAGTCTTCACCAACGG-3'
	
	RevEnd	5'-TTATGCAGGAGCCACAGAGG-3'
	
	Rev8	5'-GCCCACCTGTCGGTTCATGAAG-3'

**Figure 1:**
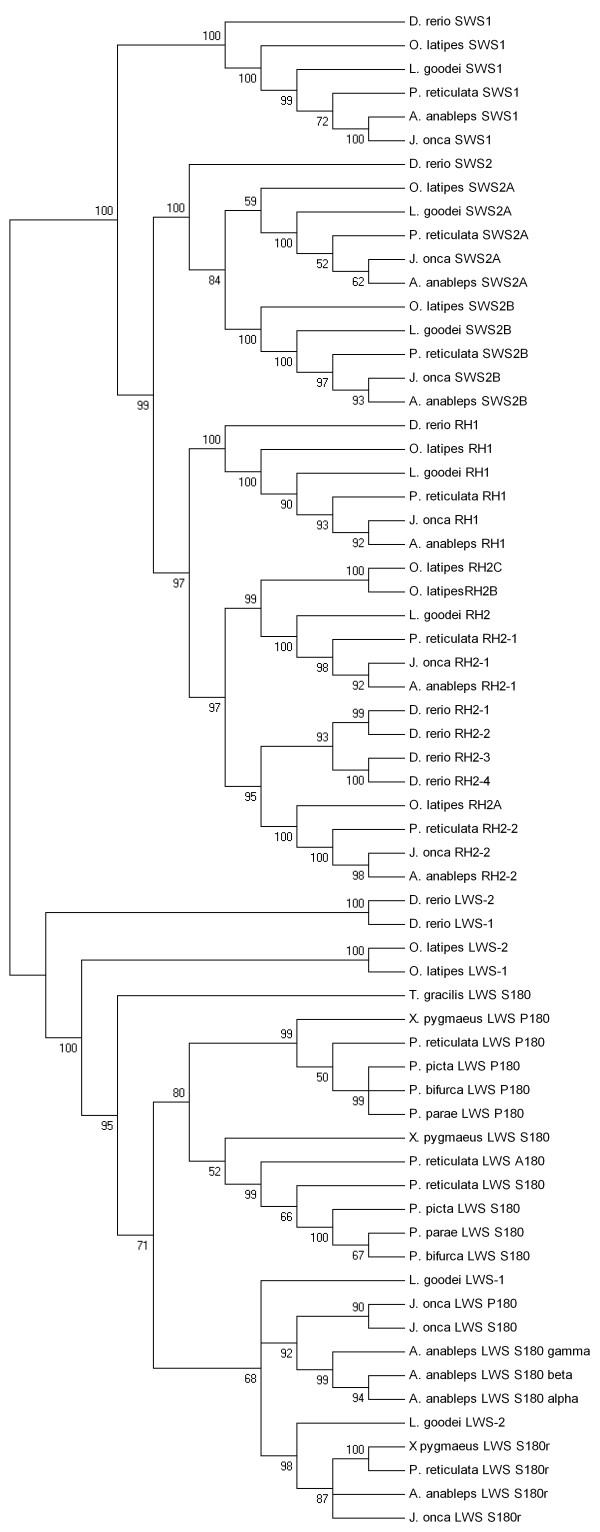
**Phylogenetic analysis of *Jenynsia onca *opsins**. A neighbour-joining bootstrap tree, which uses opsin coding sequence from *J. onca *and relatives. The percentage of trees in which the associated taxa clustered together in the bootstrap test (1000 replicates) is reported at the nodes. PAUP* 4.0B10 was used to estimate genetic distances, based on modeltest's best-fit model of evolution, and complete phylogenetic analysis [18,19] [accession numbers see Additional file 3]. All codon positions were included. Pair-wise deletion was used in the case of missing nucleotides for the analysis.

The *J. onca *opsins were aligned to orthologous sequences from other fish species. Sequences in the alignment were 573 to 930 base pairs (bp) long. We used PAUP* 4.0B10 to calculate genetic distances based on the modeltest best-fit model of sequence evolution and to reconstruct Neighbour joining (NJ) (Figure 1) and Maximum likelihood (ML) (see Additional file 1) trees [18-21]. Sequences from each opsin subfamily formed well-supported monophyletic groups, with bootstrap support ≥ 97% (1000 replicates) [22].

**Figure 2:**
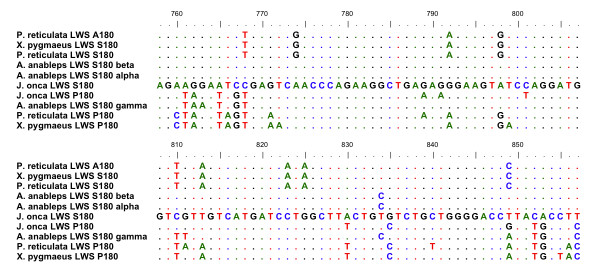
**Sequence alignment of conserved LWS P180 sequence**. A sequence alignment of the conserved 100 base pair portion (nucleotide 758 - 857) of the *J. onca *LWS P180 and *A. anableps *LWS S180γ with comparisons to Poeciliid LWS. Highlighted is the *J. onca *S180, which exemplifies the shared difference of the *J. onca *LWS P180, Poeciliid LWS P180 and *A. anableps *LWS S180γ. Accession numbers for these sequences are listed in Additional file 3.

**Figure 3:**
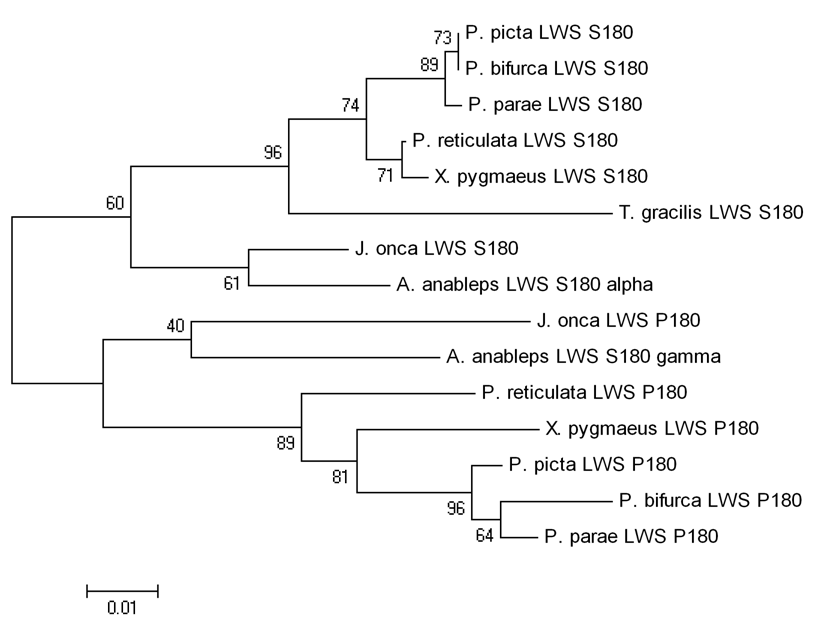
**Phylogenetic analysis of shared 3' LWS P180 sequence**. A neighbour-joining bootstrap consensus tree of a 243 base pair portion of 3' LWS S180 and LWS P180 opsins from *J. onca *and relatives. The percentage of trees in which the associated taxa clustered together in the bootstrap test (500 replicates) is reported at the nodes. The Jukes-Cantor algorithm was used and all codon positions were included [31]. Pair-wise deletion was used in the case of missing nucleotides for the analysis. Phylogenetic analyses used MEGA4 [30] [accession numbers see Additional file 3].

The phylogenetic analysis indicated that gene identity was consistent with regard to both the subfamily that the genes fell into and species taxonomy. The LWS subfamily can be further grouped into haplotype clades, however, *J. onca *LWS S180 and LWS P180 did not fall out on the tree where they would be predicted to based on haplotype identity (Figure 1). LWS P180 was found to be highly similar to *A. anableps *LWS S180γ and Poeciliidae LWS P180 only over its 3' end (Figure 2). When only the 3' region of these two genes and the Poeciliidae LWS are used in phylogenetic analysis two distinct clades with 60% bootstrap are observed separating LWS S180 from LWS P180 (including *A. anableps *LWS S180γ) (Figure 3).

We hypothesize that two recent gene conversion events explain the observed pattern. One event would have occurred between the two *J. onca *genes, from LWS S180 to LWS P180 leaving only the original 3' end of LWS P180, masking the true orthologous relationship. This conversion event in *J. onca *also explains why the sequence surrounding the proline at amino acid postion 180, which occurs before the 3' end, is very similar to the *J. onca *LWS S180. Indeed, based on the pattern of similarity it seems likely that the P180 substitution occurred independently in the Anablepid lineage. The second conversion event we suggest occurred recently in the *A. anableps *LWS S180γ, with the majority of the 5' region of this gene having been over-written by LWS S180α. This event explains the grouping of LWS S180γ with Poeciliid LWS P180 in the 3' phylogenetic tree (Figure 3) and why LWS S180α and LWS S180γ are nearly identical until the last portion of the 3' end. This conversion event also clarifies why the *A. anableps *LWS S180γ does not have a proline at the 180 amino acid site. Our hypothesis that gene conversion has influenced sequence variation among opsin paralogs is supported by an analysis using GENECONV 1.81A, which detects sequence pairs that have abnormally long continuous regions of high sequence similarity found within regions of lower similarity overall [23,24]. GENECONV detected a gene conversion event between LWS S180 and LWS P180/S180γ in *A. anableps *from the beginning of the sequence to 629 bp (p = 0.012) and in *J. onca *from the start of the sequence to 567 bp (p = 0.036), which correspond to our predicted conversion events.

The only alternative explanation for this pattern of evolution would be convergent evolution of this 3' region (Figure 2), however given the previous observations of gene conversion in opsins it is not the most likely explanation. Homogenized key site haplotypes have been observed not only in fish, such as the guppy where the LWS A180 is a product of duplication followed by partial gene conversion, but also in humans where conversion events are often detrimental [14,25,26]. The conversion events we propose in livebearers may have been facilitated between LWS P180 and LWS S180 due to their position as tandem inverted duplicates, something that has been confirmed in both *Poecilia *and *Xiphophorous *[14].

As mentioned above, there are key sites within each opsin subclass, which may be used to estimate the opsin λ_max_. We investigated the key site haplotypes of these nine opsins and based on the LWS haplotypes we have estimated the LWS λ_max _values [27] [see Additional file 2]. The key site substitutions seen in the LWS P180 although found in other species, confer a significant change in λ_max_; the serine to proline substitution alone results in a -19 nm shift [28]. Only one key site substitution is not seen in *J. onca*'s relatives; RH2-2 deviates from the isoleucine consensus residue to a valine residue in amino acid site 65 (based on human LWS amino acid numbering [29]), although this would likely not significantly shift the λ_max _[8]. The most interesting point to glean from the haplotype comparison is that despite significant nucleotide divergence between *J. onca *and its relatives there is a large degree of amino acid conservation at key sites between orthologs.

## Conclusion

*Jenynsia onca *has nine visual opsins: three LWS, one RH1, two RH2, one SWS1 and two SWS2. Despite nucleotide level divergence between related orthologs it appears there is significant phenotypic (λ_max_) conservation with only one instance of amino acid key site residue substitution. *J. onca *in the future could help us identify differences in opsin expression that are associated with the unusual eyes of *A. anableps *and with the remarkable coloration of guppies by acting as an out-group.

## Methods

PCR primers were designed to amplify nine opsins from the five visual opsins subfamilies (Table 1). These primers were complementary to regions in each opsin gene or subfamily that were conserved in guppies (*Poecilia reticulata*), and *A. anableps*. Two primer pairs were engaged for each gene.

Each primer pair was used to survey cDNA or genomic DNA in PCR reactions using Bio-Rad iProof high-fidelity DNA polymerase in an Eppendorf™ Mastercycler^® ^EP Grad S thermocycler using the following conditions: Initial denaturation at 98°C for 30 s, 35 cycles with denaturation at 98°C for 5 s, annealing at 55 - 65°C (in 5°C intervals) for 12 s, extension at 72°C for 25 s and a final extension at 72°C for 5 min, additional primers (1 μl at 10 mM) were added, at the beginning of the last PCR cycle to prevent heteroduplex formation.

Amplicons of the predicted size were excised using QIAquick^® ^Gel Extraction Kit or purified using QIAquick^® ^PCR Purification Kit. Purified products were A-tailed using Invitrogen™ Taq polymerase and cloned using the Promega^® ^pGEM™ - T Easy Vector System II kit. Clones containing inserts of the correct size were sequenced using labelled M13 forward and reverse primers and a Licor sequencer at the University of Victoria Centre for Biomedical Research.

Live *J. onca *were obtained from a commercial supplier (The Afishionados, Winnipeg, Manitoba, Canada). Two adult *J. onca *(one male and one female) were euthanized in buffered MS222. Total RNA was isolated from the eyes using Aurum™ Total RNA Fatty and Fibrous Tissue Pack, immediately after euthanasia and enucleation cDNA was synthesized using BioRad^® ^iScript Select cDNA Synthesis Kit from total RNA. DNA was isolated from the fish carcass using QIAquick^® ^DNeasy Blood & Tissue Kit.

Two phylogenetic trees were reconstructed for the complete set of opsin sequences. The partial coding sequence tree included sequences from *Jenynsia onca, Anableps anableps, Poecilia reticulata, Xiphophorus pygmaeus, Lucania goodei, Oryzias latipes and Danio rerio*, *Poecilia picta, Poecilia parae, Poecilia bifurca and Tomeurus gracilis *sequence files used were 412 to 819 bp long. The second tree was based on 243 bp of LWS 3' coding sequence from *J. onca, A. anableps, P. reticulata, X. pygmaeus, P. picta, P. parae, P. bifurca and T. gracilis *[Accession numbers see Additional file 3]. The aligned sequences for the partial coding sequence phylogenetic tree were first used to obtain the best-fit model of evolution using Modeltest [19]. The phylogenetic reconstruction was done using ML and NJ (1000 bootstrap reanalyses) in PAUP* 4.8B10 and utilized the optimal model parameters [18,20-22]. The root of the partial coding sequence tree was positioned along the branch separating the LWS opsins from all others [30]. The 3' coding tree was constructed using MEGA4 using the Jukes-Cantor algorithm, NJ, and support for nodes were estimated using 500 bootstrap reanalyses [20,22,31,32]. Pair-wise deletion was used in the case of missing nucleotides for the analysis.

Gene conversion detection was undertaken using GENECONV version 1.81A [23]. We used the program's default values with the exception of gscale, which we set to 2. The input for analysis was a coding sequence nucleotide alignment containing *A. anableps *LWS S180α and LWS S180γ as well as *J. onca*, *X. pygmaeus*, *P. picta, P. parae, P. reticulata, and P. bifurca *LWS S180 and LWS P180.

## Competing interests

The authors declare that they have no competing interests.

## Authors' contributions

DJW & GLO carried out the PCR survey of cDNA and genomic DNA, completed the sequence alignment, and created the phylogenies. DJW drafted the manuscript. Both authors read and approved the final manuscript.

## Supplementary Material

Additional file 1 **Phylogenetic analysis of *J. onca *opsins using a Maximum-likelihood approach**. A maximum likelihood tree, which uses opsin-coding sequence from *J. onca *and relatives. PAUP* 4.0B10 was used to estimate genetic distances, based on modeltest's best-fit model of evolution, and complete phylogenetic analysis [18,19] [accession numbers see Additional file 3]. All codon positions were used and pair-wise deletion was used in the case of missing nucleotides for the analysis. Click here for file

Additional file 2 **Amino acid key site haplotypes and LWS lambda max prediction for *Jenynsia Onca *and relatives**. Key site haplotypes for *J. onca*, *A. anableps*, *P. reticulata *and *L. goodei *[Accession numbers see Additional file 3]. Amino acid site numbering is based on human LWS [29]. Lambda max values estimated from mutational analysis of other LWS opsins [27,28]. An 'X' indicates that the sequence obtained in our PCR survey did not encompass the respective site. Click here for file

Additional file 3 **Accession numbers for all sequences used**. Accession numbers of sequences generated from a degenerate PCR survey of *J. onca *and those used for phylogenetic analysis. Click here for file
