# Supplementary figures and images for: The opsin repertoire of Jenynsia onca: a new perspective on gene duplication and divergence in livebearers
Source: BMC Res Notes. 2009 Aug 5;2:159. doi: 10.1186/1756-0500-2-159 (PMC2732921; doi:10.1186/1756-0500-2-159)

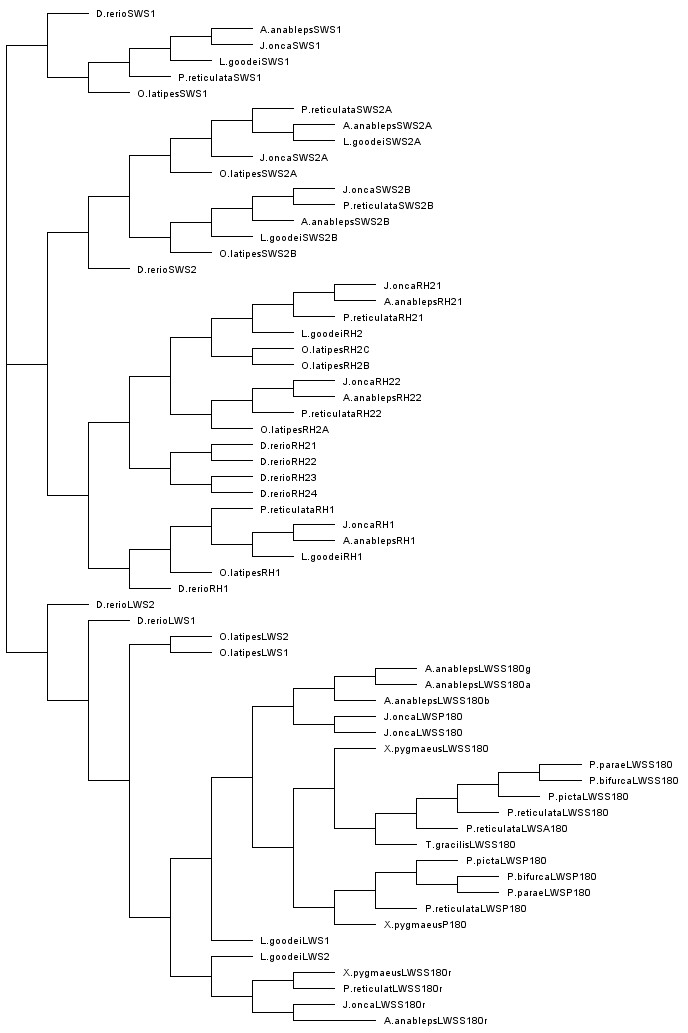

Supplement: Additional file 1 — Phylogenetic analysis of J. onca opsins using a Maximum-likelihood approach. A maximum likelihood tree, which uses opsin-coding sequence from J. onca and relatives. PAUP* 4.0B10 was used to estimate genetic distances, based on modeltest's best-fit model of evolution, and complete phylogenetic analysis [18,19] [accession numbers see Additional file 3]. All codon positions were used and pair-wise deletion was used in the case of missing nucleotides for the analysis. [file 1756-0500-2-159-S1.jpeg]
