# Supplementary material for: The opsin repertoire of Jenynsia onca: a new perspective on gene duplication and divergence in livebearers
Source: BMC Res Notes. 2009 Aug 5;2:159. doi: 10.1186/1756-0500-2-159 (PMC2732921; doi:10.1186/1756-0500-2-159)
Supplement: Additional file 3 — Accession numbers for all sequences used. Accession numbers of sequences generated from a degenerate PCR survey of J. onca and those used for phylogenetic analysis. [file 1756-0500-2-159-S3.doc]

| Scientific names | Gene name | Accession number |
| --- | --- | --- |
| *J. onca* | LWS S180 | GQ221677 |
|  | LWS P180 | GQ221676 |
|  | LWS S180r | GQ221671 |
|  | SWS1 | GQ221672 |
|  | SWS2A | GQ221673 |
|  | SWS2B | GQ221674 |
|  | RH2-1 | GQ221668 |
|  | RH2-2 | GQ221669 |
|  | RH1 | GQ221670 |
| *A. anableps* | LWS S180α | FJ11154 |
|  | LWS S180β | FJ11158 |
|  | LWS S180γ | FJ11157 |
|  | LWS S180r | FJ11155 |
|  | SWS1 | FJ11153 |
|  | SWS2A | FJ11152 |
|  | SWS2B | FJ11151 |
|  | RH2-1 | FJ11149 |
|  | RH2-2 | FJ11150 |
|  | RH1 | FJ11156 |
| *P. reticulata* | LWS S180 | EU329434 |
|  | LWS A180 | EU329442 |
|  | LWS P180 | EU329456 |
|  | LWS S180r | EU329457 |
|  | SWS1 | DQ234861 |
|  | SWS2A | FJ11159 |
|  | SWS2B | DQ234860 |
|  | RH2-1 | DQ234859 |
|  | RH2-2 | DQ234858 |
|  | RH1 | DQ912024 |
| *X. pygmaeus* | LWS S180 | EU329481 |
|  | LWS P180 | EU329478 |
|  | LWS S180r | EU329479 |
| *L. goodei* | LWS-1 | AY296740 |
|  | LWS-2 | AY296741 |
|  | SWS1 | AY296735 |
|  | SWS2A | AY296737 |
|  | SWS2B | AY296736 |
|  | RH2-1 | AY296739 |
|  | RH1 | AY296737 |
| *O. latipes* | LWS-1 | AB223051 |
|  | LWS-2 | AB223052 |
|  | SWS1 | AB223058 |
|  | SWS2A | AB223056 |
|  | SWS2B | AB223057 |
|  | RH2a | AB223053 |
|  | RH2b | AB223054 |
|  | RH2c | AB223055 |
|  | RH1 | AB180742 |
| *D. rerio* | LWS-1 | NM131175 |
|  | LWS-2 | NM001002443 |
|  | SWS1 | BC060894 |
|  | SWS2 | NM131192 |
|  | RH2-1 | NM131253 |
|  | RH2-2 | NM182891 |
|  | RH2-3 | NM182892 |
|  | RH2-4 | NM131254 |
|  | RH1 | BC05288 |
| *P. bifurca* | S180 | EU329460 |
|  | P180 | EU329465 |
| *P. picta* | S180 | EU329473 |
|  | P180 | EU329476 |
| *P. parae* | S180 | EU329468 |
|  | P180 | EU329470 |
| *T. gracilis* | S180 | EU329482 |
